# Preclinical and Clinical Evidence Supporting Use of Cannabidiol in Psychiatry

**DOI:** 10.1155/2019/2509129

**Published:** 2019-08-29

**Authors:** Gioacchino Calapai, Carmen Mannucci, Ioanna Chinou, Luigi Cardia, Fabrizio Calapai, Emanuela Elisa Sorbara, Bernardo Firenzuoli, Valdo Ricca, Gian Franco Gensini, Fabio Firenzuoli

**Affiliations:** ^1^Department of Biomedical and Dental Sciences and Morphological and Functional Imaging, University of Messina, Messina, Italy; ^2^Division of Pharmacognosy & Chemistry of Natural Products, Department of Pharmacy, University of Athens, Athens, Greece; ^3^Anesthesia, Intensive Care and Pain Therapy, A.O.U. G. Martino Messina, University of Messina, Messina, Italy; ^4^Research and Innovation Center in Phytotherapy and Integrated Medicine (CERFIT), Referring Center for Phytotherapy of Tuscany Region, Careggi University Hospital, Florence, Italy; ^5^Psychiatry Unit, Department of Health Sciences, University of Florence, Florence, Italy; ^6^Permanent Commission for Guidelines, Coordinator, Tuscany Region, Florence, Italy

## Abstract

**Background:**

Cannabidiol (CBD) is a major chemical compound present in *Cannabis sativa*. CBD is a nonpsychotomimetic substance, and it is considered one of the most promising candidates for the treatment of psychiatric disorders.

**Objective:**

The aim of this review is to illustrate the state of art about scientific research and the evidence of effectiveness of CBD in psychiatric patients.

**Methods:**

This review collects the main scientific findings on the potential role of CBD in the psychiatric field, and results of clinical trials carried out on psychiatric patients are commented. A research was conducted in the PUBMED, SCOPUS, and ScienceDirect databases using combinations of the words cannabidiol, psychiatry, and neuropsychiatric.

**Results:**

Preclinical and clinical studies on potential role of CBD in psychiatry were collected and further discussed. We found four clinical studies describing the effects of CBD in psychiatric patients: two studies about schizophrenic patients and the other two studies carried out on CBD effects in patients affected by generalized social anxiety disorder (SAD).

**Conclusion:**

Results from these studies are promising and suggest that CBD may have a role in the development of new therapeutic strategies in mental diseases, and they justify an in-depth commitment in this field. However, clinical evidence we show for CBD in psychiatric patients is instead still poor and limited to schizophrenia and anxiety, and it needs to be implemented with further studies carried out on psychiatric patients.

## 1. Introduction

**Figure 1:**
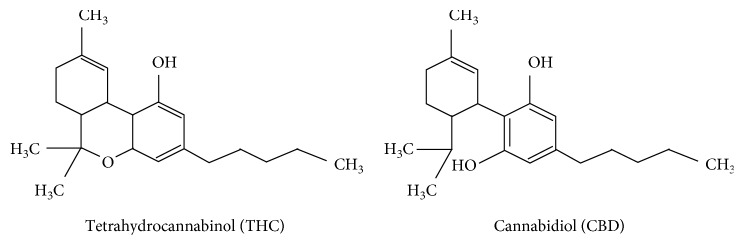
Tetrahydrocannabinol and cannabidiol chemical structure.

The plant *Cannabis* contains a complex of secondary metabolites, the so-called cannabinoids. Cannabinoids consist of more than 60 compounds of which delta-9-tetrahydrocannabinol (THC) and cannabidiol (CBD) are the most known. CBD is a resorcinol-based compound capable to mitigate the psychotomimetic effects produced by THC at high dosages [1]. CBD has no psychotomimetic effects and possesses a distinct pharmacological profile not comparable to THC that is the principal psychoactive cannabinoid-type compound in the plant of *Cannabis*. Despite the pharmacological characteristics, THC and CBD have the same chemical formula even if the atoms are differently displayed [2] (Figure 1). CBD was isolated for the first time in 1940, and its structure was described by Mechoulam et al. in 1963, while the definitive molecular configuration was established twenty-seven years later [3]. While THC acts as a partial agonist at the G protein-coupled CB1 (CB1r) and CB2 (CB2r) cannabinoid receptors, current evidence suggests that CBD does not directly interact with the endocannabinoid system (ECS) [4]. CBD has not an evident intrinsic activity over these receptors and has low-affinity for CB1r and CB2r binding [5]. Effects of CBD on intracellular signaling are widely independent of CB1 receptors [6] and the *in vivo* effects of CBD, including its anti-inflammatory properties, appear to be CB_1_-independent [7]. Anyway, the relative mechanism of action appears to be complex, and it has been not definitively studied [8].

Apart from effects involving CB1r and CB2r [9, 10], CBD modulates other cellular systems, such as the transient receptor potential subfamily V member 1 (TRPV1) cation channels [11], the orphan G-protein-coupled receptor 55 also known as GPR55 and operating as a counterpart to the standard CB1R/CB2R signaling pathway [12], fatty acid amide hydrolase (FAAH) [13], peroxisome proliferator-activated receptor gamma (PPAR*γ*) [14], serotonin 1A (5-ht1a) [15, 16], and *μ*- and *δ*-opioid [17] receptors. CBD modulates calcium flux through the control on intracellular calcium stores [18] and is a competitive inhibitor of adenosine uptake [19, 20]. Some CBD effects at these targets in *in vitro* assays only manifest at high concentrations, which may be difficult to achieve *in vivo*, particularly given CBD's relatively poor bioavailability [4]. The volume of distribution of CBD following intravenous administration is 32 l/kg. CBD reaches many organs and tissues, including the eye and the central nervous system (CNS). After hepatic hydroxylation to 7-hydroxy cannabidiol (7-OH-CBD), subsequent faecal and, to a lesser extent, urinary excretion of metabolites occurs [21].

CBD possesses anti-inflammatory and antioxidant properties and has been considered as a potentially new pharmacological approach for neuroprotection [22]. Early findings showing that CBD attenuated psychotomimetic and anxiogenic effects induced by high doses of THC in humans led to believe that this cannabinoid could possess antipsychotic and anxiolytic properties [23]. Likewise, it is remarkable that CBD is not a psychotomimetic agent since it does not produce effects related to abuse and dependence as THC does [24] but it can be effective at the CNS level, crossing the blood-brain barrier, and clinical findings suggest that CBD could represent a useful strategy for the treatment of neurological diseases such as epilepsy [25], neurodegenerative diseases such as Alzheimer's disease (AD) [26], Huntington's disease (HD) [27], and Parkinson's disease (PD) [28], and neonatal brain ischemia [29].

Several studies suggest that deficiencies of ECS can contribute to the development of psychiatric diseases [30], particularly mood disorders [31], schizophrenia [32], depression [33], and anxiety [34]. CBD is considered a safe substance and as one of the most promising candidates for the treatment of psychiatric disorders.

The aim of this review is to illustrate the state of art about scientific research and the evidence of effectiveness of CBD in psychiatric disorders.

## 2. Methods

### 2.1. Search Strategy

Using electronic databases, such as PubMed, Scopus, and ScienceDirect, the search was carried out independently by two researchers with “cannabidiol” as the main keyword starting from January 1970 to February 2019. In a second step, the words “psychiatry,” “schizophrenia,” “anxiety,” “depression,” “autism,” “anxiolytic,” “antidepressant,” and “antipsychotic” were one by one added to the keyword “cannabidiol.” Articles published on peer-reviewed scientific journals describing psychiatric CBD preclinical effects and clinical studies carried out with patients affected by psychiatric diseases were collected and discussed. Case series and case reports and human studies investigating psychiatric issues only in healthy individuals not affected by psychiatric diseases were excluded.

### 2.2. Study Selection

The authors collected all preclinical and clinical findings carried out investigating the effects of CBD alone, not in combination with other substances, in the psychiatric field. Articles describing CBD preclinical effects and four studies reporting CBD clinical effects in psychiatric patients were found. Articles were included if they met the following inclusion criteria: articles written in English language, CBD used alone, and CBD used not in combination with other substances. Studies carried out with cannabis preparations containing cannabinoids or other compounds other than CBD or products containing THC or other compounds were excluded. Involvement of CBD in neurodegenerative diseases bordering with the psychiatric field such as Parkinson's Disease and Alzheimer's Disease has been discussed in a previous review about neurological aspects of CBD use [25]. This review followed the PRISMA statement.

## 3. Results

**Figure 2:**
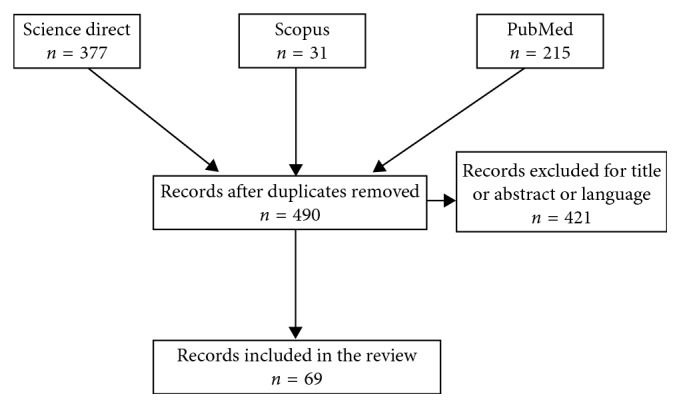
Flow chart of records identified through database searching (*n* = 623).

Preclinical and clinical studies on potential role of CBD in psychiatry were collected and further discussed. We found four clinical studies describing the effects of CBD in psychiatric patients, two studies about schizophrenic patients, and two studies carried out on CBD effects in patients affected by generalized SAD. The total number of 73 scientific articles were finally selected and discussed for the present review (Figure 2). Tables 1 and 2 summarize for each clinical study patients' pathological characteristics, study design, population, treatment, endpoints, outcome, and reference.  Table 3 reports clinical study carried out on healthy subjects. For each clinical study considered, CBD effects, study design, population, treatment, endpoints, outcome, and reference.  Table 4 reports quality assessment of randomized controlled trials by the Jadad scoring system.

### 3.1. CBD and Schizophrenia

Schizophrenia is a severe, lifelong mental disorder affecting about 1% of world's population. This pathology is characterized by positive and negative symptoms, and also the cognitive system is affected often leading to functional impairment. Unfortunately, not all patients respond to pharmacological treatments [40]. Recent findings focused on the participation of ECS in the pathogenic mechanisms of schizophrenia and suggested that CBD may have antipsychotic properties. CBD effects do not appear to depend on dopamine receptor antagonism and thus may be a promising new pharmacological approach to the treatment of this disease [41]. Suggestion for the involvement of ECS comes from studies linking the abuse of cannabis and schizophrenia and from observation of increased endocannabinoids and changes in the expression of cannabinoid receptors in several brain regions of patients affected by schizophrenia [42].

CBD has a pharmacological profile similar to atypical antipsychotics, reducing psychotic-like symptoms. CBD (15–60 mg/kg), like haloperidol (0.25–0.5 mg/kg), dose-dependently reduced stereotyped behavior induced by apomorphine in rats at doses unable to produce catalepsy [43]. Either central injection in the dorsal striatum or systemic injection of CBD (30–60 mg/kg) reduces catalepsy induced by haloperidol, thus suggesting that it can act in the dorsal striatum to improve haloperidol-induced catalepsy via postsynaptic 5-HT1A receptors [44]. CBD is also known to ameliorate hyperlocomotion and prepulse inhibition of the startle reflex deficits in acute models of schizophrenia [45].

Starting from the evidence that chronic THC exposure during adolescence in rats induces schizophrenic-like behaviors and decreases mTOR-p70S6K signaling pathway in the prefrontal cortex of adult brains, it has been shown that both CBD and THC can induce opposite functional effects within these pathways [46]. Since THC and CBD have opposite effects on the activity of the hippocampus, medial prefrontal cortex (MPC), striatum, and superior temporal and occipital cortices, it has been suggested that different patterns of brain activation could underlie their opposing actions on schizophrenia-related circuits [47].

Consistently with these findings, antipsychotic medications have been recently reported to increase mammalian target of rapamycin (mTOR) and the ribosomal protein p70S6K signaling through direct actions on dopamine D2-selective neuronal populations in the striatum. Further studies demonstrate that CBD antipsychotic effects could be due to mechanistic effects reducing dopaminergic sensitization directly within the mesolimbic pathway, considered the primary brain target for antipsychotic intervention [48]. It has been also suggested that CBD activity as dopamine partial agonist action of CBD may account for its clinical antipsychotic effects [49]. Furthermore, as well as for the antipsychotic drug aripiprazole, CBD is known to facilitate 5-HT1A receptor-mediated neurotransmission in schizophrenia-related areas, including the rodent MPC [50].

A small-scale clinical study investigating on CBD antipsychotic symptoms in humans confirmed its potential as an effective, safe, and well-tolerated antipsychotic chemical substance. This study was performed as a double-blind, randomized clinical trial comparing the effects of CBD of CBD vs amisulpride in acute schizophrenia. Both treatments appeared to be safe but with CBD showing a better side-effect profile. Antipsychotic effects of CBD were associated with a significant increase in serum anandamide levels. These results induced the authors to hypothesize that clinical improvement caused by CBD inhibition of anandamide deactivation [35] (Table 1).

More recently, CBD was investigated in a randomized double-blind parallel-group trial, on patients with schizophrenia receiving CBD (1000 mg/day; *N* = 43) or placebo (*N* = 45) alongside their existing antipsychotic medication for 6 weeks. Participants were assessed before and after treatment using psychiatric scales. After 6 weeks of treatment, CBD was well tolerated and with respect to the placebo group, the CBD group had lower levels of negative symptoms and were more likely to have been rated as improved. Patients treated with CBD also showed greater improvements that fell short of statistical significance in cognitive performance and in overall functioning [36]. Results of the study induced the authors to conclude that CBD has beneficial effects in patients with schizophrenia (Table 1).

CBD has been also evaluated for the treatment of cognitive impairments associated with schizophrenia. Cognitive, symptomatic, and side effects of oral CBD (600 mg/day) added to a stable dose of antipsychotic medication were compared versus placebo in a 6-week, randomized, placebo-controlled, parallel group, in 36 stable antipsychotic-treated patients diagnosed with chronic schizophrenia. All subjects completed the MATRICS Consensus Cognitive Battery (MCCB), and psychotic symptoms were assessed using the Positive and Negative Syndrome Scale (PANSS) at baseline and biweekly. Results showed a significant decrease in PANSS scores. Side effects were similar between CBD and placebo, excepted for sedation, which was more prevalent in the CBD group. On this basis, the authors concluded that add-on of CBD was not associated with an improvement in MCCB or PANSS scores in stable antipsychotic-treated outpatients with schizophrenia [51].

### 3.2. CBD and Anxiety

Anxiety is an adaptive, physiological mechanism essential for survival. It is characterized by a state of increased vigilance and responsiveness producing defensive behaviors with the aim to prevent or reduce harm to the organism when we face unexpected and/or potentially dangerous situations or conditions [52]. Symptoms arising from excessive anxiety are present in several psychiatric disorders, including generalized anxiety disorder (GAD), panic disorder (PD), and social anxiety disorder (SAD) [53]. Even though activation of serotonergic 5-HT1A receptor appears to mediate anxiolytic effects, CB1r also seems to be involved. CB1r activation probably mediates CBD enhancement of fear extinction, reconsolidation blockade, and prevention of the long-term adverse anxiogenic consequences of stress [52].

Studies carried out by using experimental animal models suggest a possible role of ECS in panic-like responses. In particular, it has been observed as activation of CB1r located in the periaqueductal gray reduces the escape reaction induced by stimulation of this brain region [54]. In the same manner, CB1r agonists injected either systemically or directly in the PAG also attenuate the induced escape response at elevated T-maze, a panicolytic effect [55]. Involvement of hippocampal neurogenesis in the anxiolytic effect of CBD in mice subjected to 14-days chronic unpredictable stress (CUS) has been investigated. It was observed that repeated administration of CBD (30 mg/kg i.p.) prevented the anxiogenic effect of CUS and increased hippocampal progenitor proliferation and neurogenesis in wild-type mice. According to the authors, anxiolytic effects of CBD involved CB1r, as CBD administration increased hippocampal anandamide levels and administration of the CB1r antagonist AM251 prevented CBD actions. Moreover, the same experiments showed that endocannabinoid depletion by FAAH overexpression prevented cell proliferation induced by CBD. On this basis, the authors suggested that anxiolytic effect produced by chronic CBD administration in stressed mice could be due to endocannabinoid-mediated proneurogenic action in the adult hippocampus [56].

The potential of CBD acutely or chronically administered (21 days) to reduce anxiogenic responding produced by foot shock (FS) stress 24 h prior to the LD test in rats pretreated with THC (1 mg) was investigated. Administration of CBD (5 mg/kg) prevented the FS-induced anxiogenic-like responding [57].

It has been observed that repeated (14 days) CBD injections attenuate the anxiogenic effects induced by CUS in mice. After 14 days, CBD (30 mg/kg) induced anxiolytic responses in stressed animals in the elevated plus maze and novelty suppressed feeding tests, which were blocked by pretreatment with a CB1 (AM251) or CB2 (AM630), but not by 5-ht1a (WAY100635) antagonist. These effects were associated with an increase in hippocampal neurogenesis and spine density in the dentate gyrus of the hippocampus. AM251 and AM630 abolished the effects of CBD on spine density. These findings indicate that CBD prevention of CUS-induced behavioral effects is probably caused by facilitation of endocannabinoid neurotransmission and consequent CB1r/CB2r activation, which could recruit intracellular/synaptic proteins involved in neurogenesis and dendritic remodeling [58].

Rats subjected to the spared nerve injury model for 24 days show decreased 5-HT firing activity, mechanical allodynia, and increased anxiety-like behavior in the elevated plus maze test, open-field test, and novelty-suppressed feeding test. Treatment with acute intravenous (i.v.) increasing doses of CBD (0.1–1.0 mg/kg) decreased in these animals the firing rate of 5-HT neurons in the dorsal raphe nucleus, which was prevented by administration of the 5-HT1A antagonist WAY100635 (0.3 mg/kg, i.v.) and the TRPV1 antagonist capsazepine (1 mg/kg, i.v.) but not by the CB1 receptor antagonist AM251 (1 mg/kg, i.v.). Seven days of treatment with CBD reduced and decreased anxiety-like behavior associated with mechanical allodynia and normalized 5-HT activity. Anxiolytic effect was blocked only by WAY100635. These experiments seem to confirm that reduction of anxiety associated with analgesia produced by CBD predominantly through TRPV1 activation is mediated by 5-HT1A receptor activation [59].

A clinical study was carried out to investigate the possible interaction between CBD and THC in healthy humans. Volunteers received orally placebo, THC (30 mg), CBD (15, 30, or 60 mg), and mixtures of THC plus CBD. While THC alone increased pulse rate, disturbed time tasks, and induced strong psychological reactions in the subjects, CBD alone provoked no effects. When both drugs were given together, CBD was efficient in decreasing the anxiety and blocking most of the effects of THC [39] (Table 3).

Another clinical study carried out on eight volunteers showed that CBD could reduce the anxiety caused by THC. Volunteers received orally one of the following treatments: THC (0.5 mg/kg); CBD (1 mg/kg); mixture (0.5 mg/kg THC + 1 mg/kg CBD); placebo and diazepam (10 mg). Results confirmed that CBD blocks the anxiety provoked by THC [23] (Table 3).

Two clinical studies on the effects of CBD in psychiatric patients who have been diagnosed with pathological anxiety were found. A double-blind randomized study compared the effects on anxiety induced by simulation public speaking test on healthy control patients and treatment-naÏve patients affected by social anxiety disorder (SAD) who received before the test a single dose of CBD (600 mg) or placebo. CBD significantly reduced anxiety, cognitive impairment, and discomfort in speech performance and significantly decreased alert in anticipatory speech [37] (Table 2).

Another randomized controlled double-blinded crossover study investigated CBD effects in patients with generalized SAD using neuroimaging. Regional cerebral blood flow (rCBF) at rest was measured twice using (99 m) Tc-ECD SPECT in 10 treatment-naïve patients with SAD. In the first session of the study, subjects were treated with oral CBD (400 mg) or placebo, and in the second session, a crossover procedure was applied. Compared to placebo, CBD significantly decreased subjective anxiety and reduced ethyl cysteinate dimer (ECD) uptake in the left parahippocampal gyrus, hippocampus, and inferior temporal gyrus while increased ECD uptake in the right posterior cingulate gyrus, thus suggesting an antianxiety effect of CBD in SAD probably related to its activity in limbic and paralimbic brain areas [38] (Table 2). SAD is one of the most common and impairing anxiety conditions but is poorly controlled by the currently available drugs, with only 30% of subjects achieving true remission [60]. For this reason, we need to investigate for new drugs effective against pathological anxiety. Even if there is a small number of clinical studies on patients affected by anxiety, they indicate that CBD has promising anxiolytic properties to be investigated in larger clinical studies.

### 3.3. CBD and Depression

On the basis of laboratory findings, CBD has been proposed as a putative novel antidepressant. Experimental animal studies showed CBD (200 mg/kg intraperitoneally; i.p.) antidepressant-like effects in mice subjected to the forced swimming test (FST) and also under chronic stress conditions [61]. CBD is effective in animal models of predictive of antidepressant effect. CBD promotes both a rapid and a sustained antidepressant effect in animal models. This effect seems to be due to its ability to interact with multiple neurotransmitter systems involved in depression, including the serotonergic, glutamatergic, and endocannabinoid systems. Moreover, it has been shown that CBD increases brain-derived neurotrophic factor (BDNF) levels and synaptogenesis in the medial prefrontal cortex, as well as it increases neurogenesis in the hippocampus [62]. FST is a model of behavioral despair whereby mice placed in an inescapable situation (a cylinder of water) usually exhibit behavioral despair. An antidepressant-like effect is elicited as a reduction in immobility duration and sustained escape attempts (swimming and climbing) [63]. Evaluation of systemic i.p. CBD in the FST revealed a significant decrease in immobility time significant at the dose of 200 mg/kg [61]. Other experiments showed that CBD (30 mg/kg) anti-immobility effects were comparable to those of imipramine and reduced by WAY100635, antagonist of 5-HT1A receptors. These data suggested that CBD-antidepressant-like effects are probably mediated by activation of 5-HT1A receptors [32]. This view is supported by other experiments in rats were centrally injected with CBD (10–60 nmol) in the ventral MPC, a brain area rich in serotonergic innervation and playing a significant role in stress responses. CBD significantly reduced the immobility time in the FST, and this activity was prevented by central administration of either 5-HT1A antagonist WAY100635 (10 and 30 nmol) or CB1 antagonist AM251 (10 pmol). Overall, these results indicated that the increase of CBD into the vmPFC may induce antidepressant-like effects mediated by activation of both CB1 and 5-HT1A receptors [64]. More recent laboratory findings suggest that CBD-antidepressant-like effects may be related to rapid changes in synaptic plasticity in the vmPFC through activation of the brain-derived neurotrophic factor-tropomyosin receptor kinase B (BDNF-TrkB) signaling pathway [65, 66].

Antidepressant/antianhedonic-like effects of CBD were also evaluated using the genetic “depressive-like” Wistar-Kyoto (WKY) rat model. Oral pretreatment with CBD (15, 30, and 45 mg/kg) showed a prohedonic effect on WKY rats at 30 mg/kg in the saccharin preference test. CBD also increased exploration and locomotion, indicating an increase in the motivation to explore [63, 67].

More recently, it has been shown that ineffective doses of CBD (7 mg/kg), when coadministered with ineffective doses of fluoxetine (5 mg/kg) or desipramine (2.5 mg/kg), produced significant antidepressant-like effects. Pretreatment with para-chlorophenylalanine (PCPA; 150 mg/kg, i.p., per day for 4 days) depleting central serotonin abolished CBD-antidepressant-like effects in FST, thus indicating the involvement of serotonergic mechanisms [65]. Clinical evidence of CBD-antidepressant effects in people affected by depression does not exist.

### 3.4. CBD and Autism

Autism spectrum disorders (ASDs) are a group of disabilities characterized by repetitive behavioral and activity patterns and social interaction disorders with a worldwide prevalence about 1% and prevalent in males [68]. The ECS has a role in maintaining adequate social functioning, and it seems to be affected in autism [69].

Anandamide is one of the endocannabinoids most studied for its potential implication in autism. It has been suggested that low levels of anandamide may contribute to the pathophysiology of autism [70]. A clinical study was performed through liquid chromatography-tandem mass spectrometry methodology analysis of anandamide concentration in banked blood samples collected from a cohort of 112 children with and without autism. Anandamide concentrations were significantly lower in autism cases (*N* = 59) compared to control children (*N* = 53) [71]. Finally, data from rodent autism models demonstrate genetic mutations in the neuroligin gene disrupt tonic endocannabinoid signaling [72] and the importance of its signaling mode in neurodevelopment and psychiatric disorders [67]. No clinical study investigating effects of CBD in people affected by autism has been published.

## 4. Discussion and Conclusions

Increasing evidence shows that CBD could have a role in the therapy of mental diseases. Previous scientific articles faced this argument focusing on pharmacological activity of CBD [73] and suggesting that CBD may have an effective therapeutic role in the treatment of psychiatric disorders on the base of clinical studies and case reports [73]. Results of our research, enriched in assessment of methodological quality of the studies, confirm the view of this cannabinoid as a promising molecule especially in particular sectors of psychiatry such as schizophrenia, anxiety, depression, and autism.

Even though CBD effects in brain has not yet been fully investigated, it is known that it exerts a positive impact on some neuroplasticity markers of antidepressant effects, such as increased BDNF levels [74], restores the impaired neuroproliferation caused by chronic stress in animals [56], and shows to have anti-inflammatory, antioxidant, immunomodulatory and neuroprotective effects, these probably mediated by interaction with peroxisome proliferator-activated receptor-c and stimulation of hippocampal neurogenesis [14]. CBD properties reducing inflammation and oxidative stress associated with neurotoxicity, together with the absence of psychoactive effects make CBD a candidate to be a new approach for the treatment of psychiatric disorders. Nevertheless, our review shows that despite the potential utility supported by the great number of experiments using laboratory experimental models, evidence for therapeutic effects of CBD in the psychiatric field is restricted to a few clinical studies investigating CBD in schizophrenic patients and subjects affected by anxiety. Moreover, it is noteworthy that beneficial effects are generally related to the use of high doses of CBD and its use as an adjuvant [75]. Clinical trials' quality assessment according to Jadad score shows that all the four studies were randomized and double-blinded, but the method to generate random allocation sequence is not described in three studies and the double-blinding procedure was not reported for all the studies (Table 4). However, results from these studies are promising and suggest that CBD may have a role in the development of new therapeutic strategies in these mental diseases and they justify an in-depth commitment in research in this field. In conclusion, clinical evidence for CBD in psychiatric patients is instead still poor and limited to schizophrenia and anxiety; thus, it needs to be implemented with further larger and well-designed studies. However, since it seems to have a good risk profile and as CBD effects do not appear to depend on dopamine-receptor antagonism, this cannabinoid could represent a new class of treatment drugs for psychiatric disorders.

## Figures and Tables

**Table 1:** Clinical studies investigating CBD effects in schizophrenic patients.

Psychiatric disorder	Study design	Population	Treatment	Endpoints	Outcome	Reference
Acute schizophrenia	Randomized, double-blinded, monocenter, parallel-group, controlled clinical trial	Sample: 42 acutely exacerbated schizophrenic (men and women) patients aging 18–50 years were enrolled	CBD or amisulpride starting with 200 mg per day each and increased stepwise by 200 mg per day to a daily dose of 200 mg four times daily (total 800 mg per day)	BPRS and PANSS were both used as primary outcome measures for the assessment of psychotic symptoms at baseline, days 14 and 28 of the treatment period Serum anandamide levels Side effects were evaluated using EPS, measurement of serum prolactin and body weight	CBD treatment produced clinical improvement accompanied by a significant increase in serum anandamide levels	Cannabidiol enhances anandamide signaling and alleviates psychotic symptoms of schizophrenia; Leweke et al. [35]

Schizophrenia or a related psychotic disorder	Multicenter, double-blind, randomized, placebo-controlled, parallel-group trial	Sample: 51 patients; CBD group: *N* = 43; placebo group: *N* = 45. Total sample mean age: 40.8, SD: 11.69 years	Patients were randomly assigned in a 1 : 1 ratio to receive CBD 1,000 mg/day or matching placebo alongside the existing antipsychotic medication, administered in two divided doses (morning and evening) for 6 weeks	Positive psychotic symptoms (measured using the PANSS positive subscale); Scale for the Assessment of Negative Symptoms (SANS) score; Clinical Global Impressions Scale (CGI-I); Global Assessment of Functioning (GAF) Scale score; Brief Assessment of Cognition in Schizophrenia (BACS); Carer Global Impression of Change Scale; Simpson-Angus Scale; body weight, waist measurement, BMI, and HDL cholesterol levels	The CBD group had lower levels of positive psychotic symptoms and were more likely to have been rated as improved and as not severely unwell by the treating clinician. Patients who received CBD also showed greater improvements that fell short of statistical significance in cognitive performance and in overall functioning. CBD was well tolerated, and rates of adverse events were similar between the CBD and placebo groups	Cannabidiol (CBD) as an adjunctive therapy in schizophrenia: a multicenter randomized controlled trial; McGuire et al. [36]

CBD: cannabidiol; BPRS: Brief Psychiatric Rating Scale; EPS: Extrapyramidal Symptom Scale; PANSS: Positive and Negative Syndrome Scale; PANSS is a medical scale used for measuring symptom severity of patients with schizophrenia.

**Table 2:** Clinical studies investigating CBD effects in social anxiety disorder (SAD).

Psychiatric disorder	Study design	Population	Treatment	Endpoints	Outcome	Reference
Generalized social anxiety disorder (SAD)	Randomized, double-blind, controlled trial	Sample: a total of 24 never-treated subjects with generalized SAD and 12 healthy control subjects	SAD patients were randomly assigned to two groups with 12 subjects each to receive CBD (600 mg) or placebo 1 hour and half an hour before the test Healthy controls (n. 12) did not receive any medication	Simulation public speaking test (SPST) Subjective ratings on VAMS and negative self-statement scale, and physiological measures (blood pressure, heart rate, and skin conductance) were measured at six different time points during the SPST	Pretreatment with CBD significantly reduced anxiety, cognitive impairment, and discomfort in speech performance and significantly decreased alert in anticipatory speech	Cannabidiol reduces the anxiety induced by simulated public speaking in treatment-naıve social phobia patients; Bergamaschi et al. [37]

Generalized social anxiety disorder (SAD)	Randomized, double-blind, placebo-controlled, crossover study	Sample: 10 treatment-naıve patients with SAD aging 20–33 years (mean age 24.2 years; SD 3.7)	Acute oral dose of CBD (400 mg) or placebo	Brief Social Phobia Scale (BSPS) and Social Phobia Inventory (SPIN) regional cerebral blood flow (rCBF) at rest was measured twice using (99 m) Tc-ECD SPECT	Reduction anxiety in SAD patients associated with reduced ECD uptake in the left parahippocampal gyrus, hippocampus, and inferior temporal gyrus, and increased ECD uptake in the right posterior cingulate gyrus	Neural basis of anxiolytic effects of cannabidiol (CBD) in generalized social anxiety disorder: a preliminary report; Crippa et al. [38]

CBD: cannabidiol; VAMS: Visual Analogue Mood Scale; BSPS: Brief Social Phobia Scale; SPIN: Social Phobia Inventory; Tc-99 ECD: technetium-99 m ethyl cysteinate dimer (ECD); SPECT: single photon emission computed tomography; SD: standard deviation.

**Table 3:** Clinical studies investigating on CBD anxiolytic effect in healthy subjects.

CBD effects	Study design	Population	Treatment	Endpoints	Outcome	Reference
Reduction of delta 9-THC provoked anxiety	Double-blinded, placebo-controlled clinical trial	Sample: 8 healthy volunteers (six men and two women), aged between 20 and 38 years (average 27)	Each volunteer participated in five experimental sessions, separated by a minimum interval of 1 week. Volunteers received orally one of the following treatments: Delta-9-THC 0.5 mg/kg, 1 mg/kg CBD, a mixture containing 0.5 mg/kg delta 9-THC and 1 mg/kg CBD; 10 mg placebo; 10 mg diazepam as control group	Interviews and spontaneous reports, Spielberger's state- trait anxiety inventory (STAI), Addiction Research Center Inventory for Marihuana Effects (ARCI-Mu), Analogue Self-Rating Scale for Subjective Feelings; Scale of Bodily Symptoms Radial Artery Pulse Rate was used to assess subject's anxiety state	CBD treatment blocked the anxiety provoked by delta 9-THC This antagonism does not appear to be caused by a general block of delta 9-THC effects since no change was detected in the pulse-rate measurements	Action of cannabidiol on the anxiety and other effects produced by delta 9-THC in normal subjects; Zuardi et al. [23]

Modulation of delta 9-THC effects	Randomized, double-blind, placebo-controlled clinical trial	Sample: 40 healthy male volunteers aged between 21 and 34 years	8 groups of 5 volunteers each received, respectively, placebo, 30 mg of delta 9-THC, 15, 30, and 60 mg of CBD, and mixtures of 30 mg of delta-9-THC plus either 15, 30, or 60 mg of CBD	Pulse rate, time production tasks and psychological logical reactions were measured at several time intervals after drug ingestion	The dose of 15–60 mg of CBD alone provoked no effects CBD blocked most of the effects of delta 9-THC when both drugs were given together CBD also decreased the anxiety component of Ag-THC effects	Cannabidiol modulates the effects of a 9-tetrahydrocannabinol in man [39]

CBD = cannabidiol; delta-9-THC = delta-9-tetrahydrocannabinol.

**Table 4:** Clinical trials quality assessment according to Jadad score.

Authors	Was the trial described as randomized?	Was the randomization procedure described and appropriate?	Was the trial described as double-blind?	Was the method of double blinding described and appropriate?	Was the number of withdrawals/dropouts in each group mentioned?	Jadad score
Bergamaschi et al. [37]	Yes	No	Yes	No	No	2
Crippa et al. [38]	Yes	No	Yes	No	No	2
Leweke et al. [35]	Yes	No	Yes	No	Yes	3
McGuire et al. [36]	Yes	Yes	Yes	No	Yes	4

The Jadad scoring system was used for the assessment of randomized controlled trials with the following 5 items. Was the study described as randomized (Yes = 1 point, No = 0 point)? was the randomization scheme described and appropriate (Yes = 1 point, No = −1 point)? Was the study described as double-blind (Yes = 1 point, No = 0 point)? Was the method of double blinding appropriate (Yes = 1 point, No = −1 point; if the answer of item 3 was No, item 4 was not calculable)? Was there a description of dropouts and withdrawal (Yes = 1 point; No = 0 point)?

## Abbreviations

SAD: Social anxiety disorder
THC: Delta-9-tetrahydrocannabinol
CBD: Cannabidiol
CBDr: Cannabinoid receptors
ECS: Endocannabinoid system
TRPV: Transient receptor potential subfamily V member
FAAH: Fatty acid amide hydrolase
PPAR*γ*: Peroxisome proliferator-activated receptor gamma
5-ht1a: Serotonin 1A receptor
CNS: Central nervous system
7-OH-CBD: 7-Hydroxy cannabidiol
AD: Alzheimer's disease
HD: Huntington's disease
PD: Parkinson's disease
mTOR: Mammalian target of rapamycin
MPC: Medial prefrontal cortex
GAD: Generalized anxiety disorder
PD: Panic disorder
CUS: Chronic unpredictable stress
rCBF: Regional cerebral blood flow
ECD: Ethyl cysteinate dimer
BDNF-TrkB: Brain-derived neurotrophic factor-tropomyosin receptor kinase B
WKY: Wistar-Kyoto
PCPA: Para-chlorophenylalanine
ASD: Autism spectrum disorders.

## References

[1] Todd S. M., Arnold J. C. (2016). Neural correlates of interactions between cannabidiol and Δ9-tetrahydrocannabinol in mice: implications for medical cannabis. *British Journal of Pharmacology*.

[2] Perez-Reyes M., Timmons M. C., Davis K. H., Wall E. M. (1973). A comparison of the pharmacological activity in man of intravenously administered 1368-11368-11368-1, cannabinol, and cannabidiol. *Experientia*.

[3] Mechoulam R., Shani A., Edery H., Grunfeld Y. (1970). Chemical basis of hashish activity. *Science*.

[4] Bih C. I., Chen T., Nunn A. V. W., Bazelot M., Dallas M., Whalley B. J. (2015). Molecular targets of cannabidiol in neurological disorders. *Neurotherapeutics*.

[5] Pertwee R. G. (2008). The diverse CB1 and CB2 receptor pharmacology of three plant cannabinoids: Δ9-tetrahydrocannabinol, cannabidiol and Δ9-tetrahydrocannabivarin. *British Journal of Pharmacology*.

[6] Laprairie R. B., Bagher A. M., Kelly M. E. M., Dupré D. J., Denovan-Wright E. M. (2014). Type 1 cannabinoid receptor ligands display functional selectivity in a cell culture model of striatal medium spiny projection neurons. *Journal of Biological Chemistry*.

[7] Devinsky O., Cilio M. R., Cross H. (2014). Cannabidiol: pharmacology and potential therapeutic role in epilepsy and other neuropsychiatric disorders. *Epilepsia*.

[8] Fernández-Ruiz J., González S., Romero J., Ramos J. A., Mechoulam R. (2005). Cannabinoids in neurodegeneration and neuroprotection. *Cannabinoids as Therapeutics*.

[9] Hayakawa K., Mishima K., Hazekawa M. (2008). Cannabidiol potentiates pharmacological effects of Δ9-tetrahydrocannabinol via CB1 receptor-dependent mechanism. *Brain Research*.

[10] Casarotto P. C., Gomes F. V., Resstel L. B. M., Guimarães F. S. (2010). Cannabidiol inhibitory effect on marble-burying behaviour: involvement of CB1 receptors. *Behavioural Pharmacology*.

[11] Costa B., Giagnoni G., Franke C., Trovato A. E., Colleoni M. (2004). Vanilloid TRPV1 receptor mediates the antihyperalgesic effect of the nonpsychoactive cannabinoid, cannabidiol, in a rat model of acute inflammation. *British Journal of Pharmacology*.

[12] Ryberg E., Larsson N., Sjögren S. (2007). The orphan receptor GPR55 is a novel cannabinoid receptor. *British Journal of Pharmacology*.

[13] Bisogno T., Hanuš L., De Petrocellis L. (2001). Molecular targets for cannabidiol and its synthetic analogues: effect on vanilloid VR1 receptors and on the cellular uptake and enzymatic hydrolysis of anandamide. *British Journal of Pharmacology*.

[14] Esposito G., Scuderi C., Valenza M. (2011). Cannabidiol reduces A*β*-induced neuroinflammation and promotes hippocampal neurogenesis through PPAR*γ* involvement. *PLoS One*.

[15] Russo E. B., Burnett A., Hall B., Parker K. K. (2005). Agonistic properties of cannabidiol at 5-HT1a receptors. *Neurochemical Research*.

[16] Pazos M. R., Mohammed N., Lafuente H. (2013). Mechanisms of cannabidiol neuroprotection in hypoxic-ischemic newborn pigs: role of 5HT1A and CB2 receptors. *Neuropharmacology*.

[17] Kathmann M., Flau K., Redmer A., Tränkle C., Schlicker E. (2006). Cannabidiol is an allosteric modulator at mu-and delta-opioid receptors. *Naunyn-Schmiedeberg’s Archives of Pharmacology*.

[18] Ryan D., Drysdale A. J., Lafourcade C., Pertwee R. G., Platt B. (2009). Cannabidiol targets mitochondria to regulate intracellular Ca^2+^ levels. *Journal of Neuroscience*.

[19] Ross R. A. (2009). The enigmatic pharmacology of GPR55. *Trends in Pharmacological Sciences*.

[20] Carrier E. J., Auchampach J. A., Hillard C. J. (2006). Inhibition of an equilibrative nucleoside transporter by cannabidiol: a mechanism of cannabinoid immunosuppression. *Proceedings of the National Academy of Sciences*.

[21] Lucas C. J., Galettis P., Schneider J. (2018). The pharmacokinetics and the pharmacodynamics of cannabinoids. *British Journal of Clinical Pharmacology*.

[22] Couch D. G., Tasker C., Theophilidou E., Lund J. N., O’Sullivan S. E. (2017). Cannabidiol and palmitoylethanolamide are anti-inflammatory in the acutely inflamed human colon. *Clinical Science*.

[23] Zuardi A. W., Shirakawa I., Finkelfarb E., Karniol I. G. (1982). Action of cannabidiol on the anxiety and other effects produced by *δ*^9^-THC in normal subjects. *Psychopharmacology*.

[24] Rong C., Lee Y., Carmona N. E. (2017). Cannabidiol in medical marijuana: research vistas and potential opportunities. *Pharmacological Research*.

[25] Mannucci C., Navarra M., Calapai F. (2017). Neurological aspects of medical use of cannabidiol. *CNS & Neurological Disorders-Drug Targets*.

[26] Fernández-Ruiz J., Sagredo O., Pazos M. R. (2013). Cannabidiol for neurodegenerative disorders: important new clinical applications for this phytocannabinoid?. *British Journal of Clinical Pharmacology*.

[27] Sagredo O., Ramos J. A., Decio A., Mechoulam R., Fernández-Ruiz J. (2007). Cannabidiol reduced the striatal atrophy caused 3-nitropropionic acid in vivo by mechanisms independent of the activation of cannabinoid, vanilloid TRPV1 and adenosine A2A receptors. *European Journal of Neuroscience*.

[28] Chagas M. H. N., Eckeli A. L., Zuardi A. W. (2014). Cannabidiol can improve complex sleep-related behaviours associated with rapid eye movement sleep behaviour disorder in Parkinson’s disease patients: a case series. *Journal of Clinical Pharmacy and Therapeutics*.

[29] Lafuente H., Pazos M. R., Alvarez A. (2016). Effects of cannabidiol and hypothermia on short-term brain damage in new-born piglets after acute hypoxia-ischemia. *Frontiers in Neuroscience*.

[30] McPartland J. M., Guy G. W., Di Marzo V. (2014). Care and feeding of the endocannabinoid system: a systematic review of potential clinical interventions that upregulate the endocannabinoid system. *PLoS One*.

[31] Mannucci C., Navarra M., Pieratti A., Russo G. A., Caputi A. P., Calapai G. (2011). Interactions between endocannabinoid and serotonergic systems in mood disorders caused by nicotine withdrawal. *Nicotine & Tobacco Research*.

[32] Schubart C. D., Sommer I. E. C., van Gastel W. A., Goetgebuer R. L., Kahn R. S., Boks M. P. M. (2011). Cannabis with high cannabidiol content is associated with fewer psychotic experiences. *Schizophrenia Research*.

[33] Zanelati T., Biojone C., Moreira F., Guimarães F., Joca S. (2010). Antidepressant-like effects of cannabidiol in mice: possible involvement of 5-HT1A receptors. *British Journal of Pharmacology*.

[34] Gomes F. V., Resstel L. B. M., Guimarães F. S. (2011). The anxiolytic-like effects of cannabidiol injected into the bed nucleus of the stria terminalis are mediated by 5-HT1A receptors. *Psychopharmacology*.

[35] Leweke F. M., Piomelli D., Pahlisch F. (2012). Cannabidiol enhances anandamide signaling and alleviates psychotic symptoms of schizophrenia. *Translational Psychiatry*.

[36] McGuire P., Robson P., Cubala W. J. (2018). Cannabidiol (CBD) as an adjunctive therapy in schizophrenia: a multicenter randomized controlled trial. *American Journal of Psychiatry*.

[37] Bergamaschi M. M., Queiroz R. H. C., Chagas M. H. N. (2011). Cannabidiol reduces the anxiety induced by simulated public speaking in treatment-naïve social phobia patients. *Neuropsychopharmacology*.

[38] Crippa J. A. S., Derenusson G. N., Ferrari T. B. (2011). Neural basis of anxiolytic effects of cannabidiol (CBD) in generalized social anxiety disorder: a preliminary report. *Journal of Psychopharmacology*.

[39] Karniol I. G., Shirakawa I., Kasinski N., Pfeferman A., Carlini E. A. (1974). Cannabidiol interferes with the effects of Δ9-tetrahydrocannabinol in man. *European Journal of Pharmacology*.

[40] Nucifora F. C., Woznica E., Lee B. J., Cascella N., Sawa A. (2018). Treatment resistant schizophrenia: clinical, biological, and therapeutic perspectives. *Neurobiology of Disease*.

[41] Peres F. F., Diana M. C., Levin R. (2018). Cannabidiol administered during peri-adolescence prevents behavioral abnormalities in an animal model of schizophrenia. *Frontiers in Pharmacology*.

[42] Zamberletti E., Rubino T., Parolaro D. (2012). The endocannabinoid system and schizophrenia: integration of evidence. *Current Pharmaceutical Design*.

[43] Zuardi A. W., Antunes Rodrigues J., Cunha J. M. (1991). Effects of cannabidiol in animal models predictive of antipsychotic activity. *Psychopharmacology*.

[44] Sonego A. B., Gomes F. V., Del Bel E. A., Guimaraes F. S. (2016). Cannabidiol attenuates haloperidol-induced catalepsy and c-Fos protein expression in the dorsolateral striatum via 5-HT1A receptors in mice. *Behavioural Brain Research*.

[45] Zuardi A. W., Crippa J. A. S., Hallak J. E. C. (2012). A critical review of the antipsychotic effects of cannabidiol: 30 years of a translational investigation. *Current Pharmaceutical Design*.

[46] Renard J., Krebs M.-O., Le Pen G., Jay T. M. (2014). Long-term consequences of adolescent cannabinoid exposure in adult psychopathology. *Frontiers in Neuroscience*.

[47] Bhattacharyya S., Crippa J. A., Allen P. (2012). Induction of psychosis by Δ9-tetrahydrocannabinol reflects modulation of prefrontal and striatal function during attentional salience processing. *Archives of General Psychiatry*.

[48] Renard J., Loureiro M., Rosen L. G. (2016). Cannabidiol counteracts amphetamine-induced neuronal and behavioral sensitization of the mesolimbic dopamine pathway through a novel mTOR/p70S6 kinase signaling pathway. *The Journal of Neuroscience*.

[49] Seeman P. (2016). Cannabidiol is a partial agonist at dopamine D2High receptors, predicting its antipsychotic clinical dose. *Translational Psychiatry*.

[50] Campos A. C., Moreira F. A., Gomes F. V., Del Bel E. A., Guimarães F. S. (2012). Multiple mechanisms involved in the large-spectrum therapeutic potential of cannabidiol in psychiatric disorders. *Philosophical Transactions of the Royal Society B: Biological Sciences*.

[51] Boggs D. L., Surti T., Gupta A. (2018). The effects of cannabidiol (CBD) on cognition and symptoms in outpatients with chronic schizophrenia a randomized placebo controlled trial. *Psychopharmacology*.

[52] Babaev O., Piletti Chatain C., Krueger-Burg D. (2018). Inhibition in the amygdala anxiety circuitry. *Experimental & Molecular Medicine*.

[53] Kroenke K., Spitzer R. L., Williams J. B. W., Monahan P. O., Löwe B. (2007). Anxiety disorders in primary care: prevalence, impairment, comorbidity, and detection. *Annals of Internal Medicine*.

[54] Viana T. G., Hott S. C., Resstel L. B., Aguiar D. C., Moreira F. A. (2015). Anti-aversive role of the endocannabinoid system in the periaqueductal gray stimulation model of panic attacks in rats. *Psychopharmacology*.

[55] Batista L. A., Bastos J. R., Moreira F. A. (2015). Role of endocannabinoid signalling in the dorsolateral periaqueductal grey in the modulation of distinct panic-like responses. *Journal of Psychopharmacology*.

[56] Campos A. C., Ortega Z., Palazuelos J. (2013). The anxiolytic effect of cannabidiol on chronically stressed mice depends on hippocampal neurogenesis: involvement of the endocannabinoid system. *The International Journal of Neuropsychopharmacology*.

[57] Rock E. M., Limebeer C. L., Petrie G. N., Williams L. A., Mechoulam R., Parker L. A. (2017). Effect of prior foot shock stress and Δ9-tetrahydrocannabinol, cannabidiolic acid, and cannabidiol on anxiety-like responding in the light-dark emergence test in rats. *Psychopharmacology*.

[58] Fogaça M. V., Campos A. C., Coelho L. D., Duman R. S., Guimara˜es F. S. (2018). The anxiolytic effects of cannabidiol in chronically stressed mice are mediated by the endocannabinoid system: role of neurogenesis and dendritic remodeling. *Neuropharmacology*.

[59] De Gregorio D., McLaughlin R. J., Posa L. (2019). Cannabidiol modulates serotonergic transmission and prevents allodynia and anxiety-like behavior in a model of neuropathic pain. *Pain*.

[60] Blanco C., Raza M. S., Schneier F. R., Liebowitz M. R. (2003). The evidence-based pharmacological treatment of social anxiety disorder. *The International Journal of Neuropsychopharmacology*.

[61] El-Alfy A. T., Ivey K., Robinson K. (2010). Antidepressant-like effect of Δ9-tetrahydrocannabinol and other cannabinoids isolated from *Cannabis sativa* L.. *Pharmacology Biochemistry and Behavior*.

[62] Silote G. P., Sartim A., Sales A. (2019). Emerging evidence for the antidepressant effect of cannabidiol and the underlying molecular mechanisms. *Journal of Chemical Neuroanatomy*.

[63] Cryan J. F., Page M. E., Lucki I. (2005). Differential behavioral effects of the antidepressants reboxetine, fluoxetine, and moclobemide in a modified forced swim test following chronic treatment. *Psychopharmacology*.

[64] Sartim A. G., Guimarães F. S., Joca S. R. L. (2016). Antidepressant-like effect of cannabidiol injection into the ventral medial prefrontal cortex-possible involvement of 5-HT1A and CB1 receptors. *Behavioural Brain Research*.

[65] Sales A. J., Fogaça M. V., Sartim A. G. (2018). Cannabidiol induces rapid and sustained antidepressant-like effects through increased BDNF signaling and synaptogenesis in the prefrontal cortex. *Molecular Neurobiology*.

[66] Sartim A. G., Sales A. J., Guimarães F. S., Joca S. R. (2018). Hippocampal mammalian target of rapamycin is implicated in stress-coping behavior induced by cannabidiol in the forced swim test. *Journal of Psychopharmacology*.

[67] Shoval G., Shbiro L., Hershkovitz L. (2016). Prohedonic effect of cannabidiol in a rat model of depression. *Neuropsychobiology*.

[68] Lai M.-C., Lombardo M. V., Baron-Cohen S. (2014). Autism. *The Lancet*.

[69] Karhson D. S., Hardan A. Y., Parker K. J. (2016). Endocannabinoid signaling in social functioning: an RDoC perspective. *Translational Psychiatry*.

[70] Zamberletti E., Gabaglio M., Parolaro D. (2017). The endocannabinoid system and autism spectrum disorders: insights from animal models. *International Journal of Molecular Sciences*.

[71] Karhson D. S., Krasinska K. M., Dallaire J. A. (2018). Plasma anandamide concentrations are lower in children with autism spectrum disorder. *Molecular Autism*.

[72] Földy C., Malenka R. C., Südhof T. C. (2013). Autism-associated neuroligin-3 mutations commonly disrupt tonic endocannabinoid signaling. *Neuron*.

[73] Premoli M., Aria F., Bonini S. A. (2019). Cannabidiol: recent advances and new insights for neuropsychiatric disorders treatment. *Life Sciences*.

[74] Magen I., Avraham Y., Ackerman Z., Vorobiev L., Mechoulam R., Berry E. (2010). Cannabidiol ameliorates cognitive and motor impairments in bile-duct ligated mice via 5-HT1A receptor activation. *British Journal of Pharmacology*.

[75] Crippa J. A. S., Zuardi A. W., Hallak J. E. C. (2010). Therapeutical use of the cannabinoids in psychiatry. *Brazilian Journal of Psychiatry*.

